# FEBAIR microsurgical principles in meningiomas and recurrence: a long-term follow-up series

**DOI:** 10.3389/fonc.2026.1761632

**Published:** 2026-05-28

**Authors:** Carlos Eduardo da Silva, Tamara Vidaletti, Paulo Eduardo Peixoto de Freitas

**Affiliations:** 1 Centro de Neurologia e Neurocirurgia - CNNc, Hospital Ernesto Dornelles, Porto Alegre, Rio Grande do Sul, Brazil; 2 Anatomy, DCBS, Universidade Federal de Ciências da Saúde de Porto Alegre, UFCSPA, Porto Alegre, Rio Grande do Sul, Brazil

**Keywords:** meningiomas, microneurosurgery, recurrence, simpson grade, tumor resection

## Abstract

**Objective and background:**

Meningiomas are the most prevalent type of primary intracranial tumor. It is essential to conduct an extensive follow-up period to assess the effectiveness of any treatment method. In this study, we present a long-term follow-up series (average ~14 years) of surgical treatments for meningiomas and evaluate the aspects related to their recurrence.

**Methods:**

This is a retrospective study focusing on surgeries performed on 99 patients with 102 meningiomas. The study reviews the patients’ medical records, operative reports, radiologic examinations, and follow-up data. The surgeries aimed to achieve the most complete and safest removal of the meningiomas, considering the patients’ medical conditions and comorbidities. Postoperative CT and MRI scans were obtained for all patients during the follow-up period.

**Results:**

The group comprised 79 women and 20 men with a median age of 56 years (range 27–87 years). The mean follow-up period was 171 months (120–280 months). Simpson grades zero, I and II resections were obtained in ~83%. Twenty cases presented recurrence during the follow-up period (~20%). In the non-recurrent group of patients (n=82), Simpson grades “zero, “ I, and II were obtained in 86.6% of cases. In the recurrent group (n=20), Simpson grades III and IV were obtained in 80% of cases. Meningiomas were WHO grade 1 in 95.1% of cases, 2 in 3.9%, and 3 in ~1%. Eight recurrent cases (40%) were originally giant meningiomas (>5cm). Previously irradiated accounted for 7% of cases. Mortality rate during the follow-up period was 7%. The Karnofsky Performance Status (KPS) score of 100 and 90 was 87%.

**Conclusions:**

This retrospective series has shown that the recurrence of meningiomas after surgery depends on the Simpson grade of resection, which is linked to incomplete tumor removal. The principles of meningioma surgery can help achieve a higher grade of resection while preserving patients’ quality of life. Long-term follow-up is essential to verify the effectiveness of any treatment method for meningiomas, and proper surgical treatment provides a high rate of cure and control over 10 years. This retrospective series has determined that the recurrence of meningiomas after surgery depends on the Simpson grade of resection. Technical principles can help improve the extent of resection during surgery while maintaining patients’ quality of life, thereby lowering the risk of recurrence. Using advanced microsurgical techniques in combination with new technologies is essential to achieving these outcomes.

## 1 Introduction

Meningiomas are the most common type of primary intracranial tumor, accounting for nearly 39% of all brain lesions (1). They are frequently found in women and older adults.1, 2 Several factors can influence the recurrence of meningiomas, including their World Health Organization (WHO) grade, cytogenetic abnormalities, molecular signature, location, and extent of resection (1–5). It is essential to have an extended follow-up period to assess treatment effectiveness, as longer follow-up is associated with lower tumor control rates. Few studies offer long-term follow-up data for the surgical management of meningiomas. This article presents a series with an average follow-up of over fourteen years and discusses factors related to meningioma recurrence.

## 2 Methods

### 2.1 Clinical and image evaluation

The local research ethics committee at Hospital Ernesto Dornelles approved the study. Patient information was de-identified before analysis. The study was conducted retrospectively on surgeries performed between 2001 and 2014, including 107 patients with meningiomas who underwent surgery by a single surgeon, C.E.S. All cases included preoperative contrast-enhanced computed tomography (CT) and magnetic resonance imaging (MRI). Bone invasion was assessed using bone window CT scans. Only patients with confirmed histopathological meningiomas were included. Data from each surgical procedure were reviewed, focusing on the Simpson grade of resection and tumor location. Simpson grade “zero” resection was defined as removing a 2 cm margin around the meningioma’s dural attachment. Medical records, operative reports, radiologic examinations, and follow-up information were also examined. Surgeries aimed for the most extensive and safe removal possible, considering each patient’s medical condition and comorbidities. Postoperative CT and MRI were obtained during follow-up in all cases. A Chi-square test assessed the association between Simpson grade resections and recurrence. The 95% confidence interval for recurrence was reported. A Binomial test evaluated whether recurrence occurred equally across techniques.

## 3 Results

Between 2001 and 2014, 107 patients underwent surgery to remove 110 meningiomas. Eight patients were excluded for follow-up loss during the period. A group of 99 patients and 102 meningiomas were included in the present study. The mean follow-up period was 171 months (120–280 months). The 102 meningiomas in the sample included the following types: 19 convexity meningiomas, 9 parasagittal, 10 sphenoid wing meningiomas (SWM), 5 spheno-orbital meningiomas (SOM), 6 clinoidal, 4 cavernous sinus, 11 olfactory groove meningiomas (OGM), 7 tuberculum sellae meningiomas (TSM), 1 temporal floor, 5 tentorial, 3 cerebellopontine angle (CPA), 16 petroclival meningiomas (PCM), 3 foramen magnum, and 3 intraventricular. The group comprised 79 women and 20 men with a median age of 56 years (range 27–87 years). Simpson grades zero, I and II resections were obtained in ~83% of the surgeries. **Table 1** presents the Simpson grade resection of the series. Twenty patients presented recurrence during the follow-up period (20.2%). In the non-recurrent group of patients (n=79), Simpson grades “zero, “ I, and II were obtained in 86.6% of cases. In the recurrent group (n=20), Simpson grades III and IV were observed in 80% of cases. Meningiomas were classified as World Health Organization (WHO) grade 1 in 95.1% of cases, 2 in 3.9%, and 3 in ~1%.

**Table 1:** Simpson grade resection performed on 102 meningiomas at the department.

Simpson grade	n	%
“Zero”	2	2.0
I	36	35.3
II	47	46.1
III	10	9.8
IV	7	6.8

The 102 meningiomas in the sample included the following types: 19 convexity meningiomas, 9 parasagittal, 10 sphenoid wing meningiomas (SWM), 5 spheno-orbital meningiomas (SOM), 6 clinoidal, 4 cavernous sinus, 11 olfactory groove meningiomas (OGM), 7 tuberculum sellae meningiomas (TSM), 1 temporal floor, 5 tentorial, 3 cerebellopontine angle (CPA), 16 petroclival meningiomas (PCM), 3 foramen magnum, and 3 intraventricular.

Recurrent meningiomas previously operated on in another department accounted for 85% of cases, and the recurrence rate in first-time surgery at our department was ~3%. Eight recurrent cases (40%) were originally giant meningiomas (>5cm), and 6 cases presented median size (3 to 5 cm). Previously irradiated in another department accounted for 7% of cases. Mortality rate during the follow-up period was 7%. The Karnofsky Performance Status (KPS) score during the follow-up period of 100 and 90 was 87%.

**Table 2** shows the distribution of the cases and recurrence by topography. **Table 3** illustrates the relation among topography, Simpson grade resection, and recurrence in 18 patients. **Table 4** relates the Simpson grade rates and recurrent groups. **Table 5** compares the original surgical locations with recurrence.

**Table 2:** Distribution of the cases and recurrence by topography.

Topography	N – total of tumors 102 cases	N – total of recurrence 20 cases (total 100%)
Convexity	19	0
Parasagittal	9	2 (10)
Intraventricular	3	0
Olfactory Groove	11	5 (25)
SOM	5	4 (20)
SW	10	3 (15)
Clinoidal	6	0
TS	7	0
Temporal floor	1	0
Petroclival	16	3 (15)
Foramen Magnum	3	0
CPA	3	1 (5)
Cavernous Sinus	4	1 (5)
Tentorial	5	1 (5)

SOM, spheno-orbital meningioma; SW, sphenoid wing; TS, tuberculum sellae; CPA, cerebello-pontine angle.

**Table 3:** Correlation between the topography, Simpson grade, and recurrence in the 20 patients.

Topography	Simpson grade I	Simpson grade II	Simpson grade III	Simpson grade IV
Olfactory Groove	1	1	3	0
SW	1	0	2	0
SOM	1	0	1	2
Tentorial	0	0	1	0
Petroclival	0	0	3	0
Cavernous Sinus	0	0	0	1
Parasagittal	0	0	1	1
CPA	0	0	1	0
RECURRENCE (%)	3 (15)	1(5)	12 (60)	4 (20)

SOM, spheno-orbital meningioma; SW, sphenoid wing; CPA, cerebello-pontine angle.

**Table 4:** Simpson grade removal in the recurrent group.

Simpson grade	Recurrent meningiomas (%) N=20
“ZERO”	0(CI95%0-77.6)
I	3 (9.4) (CI95%2.4-23.4)
II	**1 (2.4) (CI95%0.1-12.9)**
III	**12 (66.7) (CI95%41.0-86.7)**
IV	4 (44.4) (CI95%13.7-78.8)
Chisquare test p<0.001	

Bold values are statistically significant.

**Table 5:** Association between the original location of surgeries and the recurrence observed in patients with 20 recurrent meningiomas.

Topography	Our department recurrence FEBAIR principles (%)	Other department recurrence (%)	P-value
Olfactory Groove	1 (16.7)	5 (83.3)	0.219
SW	1(25)	3 (75)	0.250
SOM	0 (0)	3 (100)	0.250
Tentorial	0 (0)	1 (100)	–
Petroclival	1 (33.3)	2 (66.7)	0.999
Cavernous sinus	0 (0)	1(100)	–
Parasagittal	0 (0)	1(100)	–
CPA	0 (0)	1(100)	–
TOTAL (%)	3 (15)	17 (85)	**0.003**
Binomial test			

Bold values are statistically significant.

## 4 Discussion

Various factors contribute to the recurrence of meningiomas in any treatment approach, but the length of follow-up is crucial for assessing treatment effectiveness. The molecular signature of the tumors, which are related to their topographies, is extremely important for predicting the risk of recurrence. Despite recent molecular advancements, surgery remains the most effective method for treating meningiomas, and the extent of resection directly influences the chance of cure and disease control tumors (3, 5–7). However, only a few studies in the literature have follow-up periods longer than ten years, and this series of 99 patients, who were followed for an extended period, helps to understand the evolution of such patients. A study conducted by Jääskeläinen involving 69 patients who were followed for 20 years revealed that 19% of them experienced a recurrence of their tumors (8). The risk factors associated with recurrence were coagulation of the dura, soft tumor consistency, and bone invasion. It’s important to note that the criteria used to diagnose recurrence were limited by the available neuroimaging technology at the time of the study, which could have been a critical bias. Additionally, most of the recurrences were detected during operative procedures in patients with symptomatic tumors, which suggests that patients with small recurrent lesions might have been missed. Mansouri et al. observed a 30% global recurrence rate after 100 months (9). Colli et al. observed a total recurrence of 16.1%, with recurrence-free estimates of 83.4%, 51.6%, and 30.7% in 10, 20, and 30 years, respectively (10).

Interestingly, in the present study, which included patients treated with microsurgical and skull base techniques and diagnosed using CT scan and MRI modalities, 20% of patients experienced some recurrent disease within a median of 14 years. According to the KPS, most patients maintained their quality of life, with 87% achieving independent and autonomous living.

The present study highlights a limitation: the lack of a Kaplan-Meier curve due to the inability to obtain precise dates for surgeries performed outside our department, which could introduce bias into the analysis. There is a strong statistical correlation between greater surgical radicality and lower tumor recurrence. Simpson grades III–IV show very high recurrence rates, whereas Simpson grades I–II demonstrate low recurrence. The chi-square test yielded p < 0.001, confirming the significance of the differences among the groups. Although the individual topographic analyses did not reach statistical significance due to the small sample size, the overall analysis showed that our department had a significantly lower recurrence rate (15% vs. 85%, p = 0.003). These findings suggest that the systematic adoption of the specific surgical principles may be associated with a lower rate of meningioma recurrence. Nevertheless, single-center data and a series performed by a single surgeon introduce important biases that hinder the generalizability of the study’s results to broader practice. The fact that each institution has different patient selection criteria, surgical techniques, and protocols is another significant limiting bias.

### 4.1 Surgical principles in meningioma surgery and recurrence – FEBAIR

Surgical removal is the most effective treatment for meningiomas, and it is crucial to follow specific principles during the planning and execution of meningioma surgery, particularly given their distinct pathological findings, molecular characteristics, and natural behavior (2, 3, 11–14). We highlight these surgical principles using the acronym FEBAIR (11, 15): F- First-time surgery is the essential principle in total removal and disease control because of the preservation of anatomical structures (2, 3, 11–14, 16–21). E- Exposure considering all the limits related to the origin and extent of the tumor to choose the best approach (11). B- Bone removal of any hyperostosis adjacent to the meningioma, which indicates tumor involvement in most cases (4, 11, 13). A- Arachnoid plane dissection, attachment attack of the tumor (2, 3, 11–14, 16–21). I- Irrigation with warm saline helps identify the arachnoid plane, avoiding bipolar coagulation (11). R- Reconstruction is the final step to prevent CSF fistulas and aesthetic disturbances (2, 3, 11–14, 16–21). These principles were observed in the surgical treatment of the patients operated on in our department.

**Table 5** shows the link between the original surgical location and tumor recurrence in patients with recurrent meningiomas. Of the cases with their first surgeries at our hospital, 3 (15%) recurred during follow-up, while 17 (85%) were treated elsewhere (p=0.003). To achieve higher Simpson grade resections and lower recurrence rates while maintaining patients’ quality of life, it is crucial to carefully consider the technical aspects of surgery to ensure the most complete resection possible (11, 22–26).

### 4.2 Topography, Simpson grade, and recurrence

The location of the meningioma is a critical factor in predicting its recurrence. Tumors located on the convexity have higher rates of complete surgical removal than those that involve the venous sinuses or skull base. However, convexity tumors tend to have a higher recurrence rate due to their molecular signature biological behavior compared to cranial base lesions (9, 27, 28).

The data analysis of the present study indicated that patients with recurrent meningiomas achieved Simpson grades III and IV during their initial surgery. (**Table 4**) This finding is consistent with multiple studies in the literature (9, 14, 29–34). This factor might have caused small residual meningiomas to be left in “grade III” resections performed in other departments, which account for 85% of the recurrent cases in this study. It is important to note that meningiomas can cause hyperostosis due to tumor infiltration of the bone (25, 26). A previous surgical report used “Simpson grade III” in other departments for skull base lesions with hyperostosis that were not removed. This detail is important for predicting recurrence.

Preoperative embolization of meningiomas is not routinely performed in our department. For convexity lesions, vascular control is established early during scalp and craniotomy procedures. In skull base lesions, the extradural step is crucial for managing tumor blood supply and preventing excessive bleeding during the intradural step. Embolization has selective application and is only used in a few exceptional cases.

The recurrent cases of skull base were associated with certain aspects, which are described below:

#### 4.2.1 Olfactory groove meningiomas

There were many recurring tumors in the olfactory groove area (**Table 2**).

Of the five cases of meningiomas that recurred, three (60%) were originally giant tumors (>5cm), while the other two were medium-sized (3-5cm). This is important because giant meningiomas in the olfactory groove tend to spread laterally over the orbital roof and the floor of the anterior fossa. In our department, one case was treated primarily, while the other four had been previously operated on at different institutions.

In our department, we operated on one patient with a large meningioma using a bifrontal craniotomy. The patient experienced a recurrence and needed a more extensive removal. We treated this case with the same bifrontal approach, which involved removing the dura of the anterior cranial base and the origin of the cerebral falx at the crista Galli. In the other four cases, postoperative examinations after their first operation showed a less extensive removal, where the dura and hyperostotic infiltrated bone were preserved. This resulted in tumor recurrence, especially in one case where the recurrence was entirely in the rhinopharynx with hyperostosis in the olfactory groove.

The size of the OGM directly relates to the chance of its recurrence after treatment. Therefore, removing the tumor mainly through a transcranial approach instead of an endonasal route is essential, as the latter is only appropriate for treating small midline lesions (16, 21).

Currently, we recommend the pterional approach, which uses a larger, well-vascularized pericranial flap for cranial base reconstruction and is safer, quicker, and more practical. When treating giant meningiomas, the pterional or cranio-orbital approaches can improve the chances of completely removing the tumor and lowering the risk of recurrence. In rare cases of giant OGM, we use a bilateral transbasal approach.

González-Darder et al. reported a case series of 35 patients with only one recurrence during a medium follow-up of 55 months (35). Fountas described an 11.8% recurrence rate in olfactory groove meningiomas followed for over 5 years (33).

#### 4.2.2 Spheno-orbital meningiomas

SOMs were the second most common recurrent cases in the series, with residual bone disease being the primary cause in all cases. Three cases were operated on in another department, while one case was initially treated in our department. In all but one case, image analysis indicated that the tumor’s intradural portion had been adequately removed. However, significant remaining disease persisted in the hyperostotic bone of the greater sphenoid wing, lateral orbital wall, anterior clinoid, and temporal floor. Even in the recurrent case treated in our department, where total removal of bone and dura was assumed, the recurrence occurred ten years later in the lateral orbital wall and the intraorbital tumor portion.

To treat SOM, the key challenge is determining how much bone to remove. A CT scan with 3D reconstruction and careful preoperative planning can assist with extensive removal of the bone during the extradural step surgery (11, 16, 18, 21, 36–38). Bloss and colleagues found that the recurrence of sphenoid wing meningiomas showed a direct correlation with Simpson grade in their series (39). Ouyang et al. observed the same correlation (40).

#### 4.2.3 Petroclival

The three cases of petroclival meningiomas had originally been giant tumors but were completely removed during surgery. However, they recurred in the tentorial and clival regions, which are difficult to remove or treat radically. During the follow-up period, 13 petroclival meningiomas (including 6 original giant tumors) did not recur on MRI scans. Li et al. observed a recurrence rate of ~24% in petroclival meningiomas with clear relation to the grade of removal over a long-term follow-up period (34). Almefty et al. reported a 20% recurrence rate in their series of 64 meningiomas located at the petroclival region. They also found a correlation with subtotal removal, as observed in other series (14, 41–43). A multicenter retrospective study found that subtotal removal combined with radiotherapy provided the same control rates as gross total removal in petroclival meningiomas (44).

According to the EANS skull base section’s consensus on petroclival meningiomas, small PCM with progression can be treated with SRS. The decision process should take into account the patient’s clinical presentation and age. For medium and large PCMs, SRS is only recommended for patients who are advanced in age or have significant comorbidities that make them unsuitable for surgical treatment (45). A retrospective study indicates that grade 1 meningiomas, which were irradiated after a subtotal resection, had a 28% risk of transforming into malignant tumors, compared to 1% in those simply observed. The mortality rate was 26% for the irradiated group versus 4% for the non-irradiated group over a mean follow-up of approximately 128 months (46).

#### 4.2.4 Cavernous sinus

Cavernous sinus meningiomas are usually slow-growing tumors. These tumors tend to have a favorable natural history, and cranial nerve neuropathy can often be treated with corticosteroid therapy. In our department, four cases were operated on: one was Simpson grade II (lesion of the outer layer of the cavernous sinus), another was grade III, and the third involved partial removal due to ICA stenosis that was adherent to the tumor. The recurrent lesion in the series had undergone previous partial surgery outside our department and was classified as Simpson grade IV. The residual lesion remained stable during follow-up. Primary CS meningiomas and secondary CS invasion from other topographies are distinct tumor groups. Primary CS meningiomas can adhere to the ICA, leading to compression of the artery, invasion of the adventitia, and are associated with a higher risk of arterial rupture during removal. The feasibility of extensive removal of primary meningiomas in the CS depends on the absence of stenosis in the internal carotid artery caused by the tumor (43, 47–49). Secondary cavernous sinus meningioma invasion displaces the parasellar space, and the natural barriers of the superior, lateral, and posterior cavernous sinus walls assist in dissection and neurovascular preservation (11, 47, 50, 51). As with primary CS meningiomas, the main limitation is the invasion or stenosis of the ICA. The cranial nerves can be safely dissected and preserved, and they show high recovery rates in cavernous sinus meningiomas removal (11, 51).

### 4.3 WHO grade, cytogenetic, and molecular findings related to meningiomas recurrence

The molecular signature is essential for predicting the biological behavior of meningiomas and their likelihood of recurrence. The number of genetic abnormalities in meningiomas correlates with their biological behavior and the risk of recurrence. Even in WHO grade 1 meningiomas, recent studies have emphasized the importance of genetic alterations (5, 6). Specific changes related to certain chromosomes, such as loss of chromosome 1p, 3p, 4, 6, 10, 14q, 18, 19, or CDKN2A, are highly correlated with aggressive biological behavior (6).

The meningiomas located at the skull base exhibit a slower-growing biological behavior, which is related to their specific molecular signatures (7). In the current series, the follow-up period was quite long, and there was a gap without cytogenetic analysis. As a result, only 18 cases were included in the study regarding diagnosis. Of these, 3 had abnormal karyotypes. Two of these patients had WHO grade 2 tumors with recurrence, located in parasagittal topographies, and had undergone subtotal removal. The third case was a WHO grade 1 petroclival meningioma, with a Simpson grade II removal, which did not recur during the follow-up period. A study found that in higher-grade meningiomas, only 9% of the molecular abnormalities are shared across samples from the same tumor. This highlights the challenge of developing targeted therapies for these tumors and underscores the importance of complete removal to manage the disease (5). WHO grades 2 and 3 in meningiomas are associated with higher recurrence rates and lower progression-free survival. Almeida et al. found that all cases of recurrent meningiomas with grades 2 and 3 resulted in death due to complications related to their tumors (52). According to Colli et al., 75% of WHO grade 2 and 100% of grade 3 parasagittal meningiomas recurred in their study (53).In the present study, WHO grade 2 (3 cases) showed one recurrence (33.3%), a parasagittal meningioma with a previous Simpson grade III resection performed in another department. The single case of WHO grade 3 operated on in the series was a sphenoid wing meningioma with a Simpson Grade I resection and had no recurrence during the follow-up period. Preclinical molecular profile analysis and radiomics could be valuable and should be considered as potential options to assist in preoperative surgical decisions (54). Intraoperative Raman imaging is also beneficial when available and can aid surgeons in decision-making (55).

### 4.4 Alternative therapeutic strategies for recurrent meningiomas

The treatment options in the literature for recurrent meningiomas include observation for asymptomatic cases, surgical removal, surgical treatment combined with stereotactic radiosurgery (SRS), and SRS or other radiotherapy modalities used as standalone treatments treatment (4, 11, 21, 28, 56–58).

Multiple studies suggest that SRS is a viable treatment option for meningiomas, particularly those in the skull base, and provides effective long-term control. Radiotherapy is more effective for high-grade meningiomas and those with specific molecular signatures. A multicenter study described that a gene expression biomarker can identify meningiomas that may benefit from postoperative radiotherapy (36). However, for WHO grade 1 meningiomas, which show favorable molecular signatures such as midline and parasellar meningiomas, the natural progression of the disease presents a significant challenge in determining the true role of SRS in controlling the disease (5, 6). A recent systematic review has demonstrated that controlling the progression-free survival intervals in SRS has similar success rates to the natural history of cavernous sinus meningiomas in 10 years (28, 56–58). A recent retrospective analysis indicates that grade 1 meningiomas subjected to irradiation subsequent to subtotal resection exhibited a 28% risk of malignant transformation, in contrast to a 1% risk among those merely observed. Mortality rates were 26% in the irradiated cohort versus 4% in the non-irradiated group over an average follow-up period of approximately 128 months (59).

Currently, we observe small recurrent meningiomas in the skull base and in asymptomatic patients. For recurrences in the convexity and parasagittal regions, we recommend surgical resection. In cases of WHO grade 2 recurrent meningiomas, surgical removal should be performed promptly. For WHO grade 3 meningiomas, we treat them with SRS after initial surgery and consider surgery and radiotherapy for tumor recurrences (4, 11, 28, 36,61).

Future studies focusing on molecular advances in targeted therapy for meningiomas could provide important alternative or complementary treatment options, potentially reducing recurrence rates, especially in grade 2 and 3 lesions.

## 5 Conclusions

This retrospective observational study found that meningioma recurrence after surgery depends on the Simpson resection grade, which is associated with incomplete tumor removal. The principles of meningioma surgery can guide surgeons to achieve a higher grade of resection while maintaining patients’ quality of life, thereby decreasing the chances of recurrence. Using advanced microsurgical techniques along with new technologies is essential for achieving such outcomes. It’s important to note that long-term follow-up is necessary to confirm the effectiveness of any treatment method for meningiomas, and proper surgical treatment offers a high rate of cure and control over 10 years.

## Data Availability

Publicly available datasets were analyzed in this study. This data can be found here: N/A.
